# 455. Changing epidemiology of SARS-CoV-2 testing, positivity rates, and variant distribution in children and adults over multiple pandemic waves in New York City

**DOI:** 10.1093/ofid/ofad500.525

**Published:** 2023-11-27

**Authors:** Priya Velu, Charlene Thomas, Sophie Rand, Melissa Cushing, Zachary Grinspan, Erika Abramson, Karen P Acker, Eddie Imada, Claudio Zanettini, Luigi Marchionni, Jin-Young Han

**Affiliations:** Weill Cornell Medicine, New York, New York; Weill Cornell Medicine, New York, New York; Weill Cornell Medicine, New York, New York; Weill Cornell Medicine, New York, New York; Weill Cornell Medicine, New York, New York; Weill Cornell Medicine, New York, New York; Weill Cornell Medicine/New-York Presbyterian, New York, New York; Weill Cornell Medicine, New York, New York; Weill Cornell Medicine, New York, New York; Weill Cornell Medicine, New York, New York; NYP/Weill Cornell Medicine, New York, New York

## Abstract

**Background:**

Children and adults have had different experiences during the COVID-19 pandemic, especially in the context of new SARS-CoV-2 variants and changing vaccine eligibility. We aimed to compare the changing epidemiology of SARS-CoV-2 testing, positivity, and variants in adults versus children over multiple pandemic waves in a multi-hospital health system in New York City.

**Methods:**

We analyzed SARS-CoV-2 RT-PCR testing data from 10/1/20 to 9/11/22 from children (0-21 years) and adults ( > 21 years) and compared positivity rates during 5 pandemic waves in New York City: Wave 2 (10/1/20-6/30/21), Wave 3 (7/1/21-12/1/21), Wave 4 (12/2/21-3/5/22), Wave 5 (3/6/22-6/12/22), and Wave 6 (6/13/22-9/11/22). The first test per patient per wave was included. If a patient had a positive test, the first positive test was included. Whole genome sequencing was performed on a subset of nasopharyngeal specimens with Ct values < 33 from 12/1/20 to 5/22/22.

**Results:**

From 10/1/20 to 9/11/2022, 243,457 adults and 33,298 children were tested for SARS-CoV-2 with 15095 (6.2%) adults and 1961 (5.9%) children testing positive. Distribution of cases, positivity rates, and vaccine coverage over time are presented in Figure 1. Positivity rate was higher in adults compared to children in Wave 2 (adults 6.1%, children 4.5%, p< 0.001), similar in Wave 3 (adults 2.4%, children 2.2%, p = 0.2), higher in children in Wave 4 (adults 12%, children 16%, p< 0.001) and similar in Wave 5 (3.5%, 3.8%, p = 0.6) and Wave 6 (6.8%, 7.2%, p = 0.7). In Wave 4, the high positivity rate in children was driven by younger age groups, outpatient testing, and unvaccinated children (Figures 2-4). WGS of 1996 adult and 381 pediatric SARS-CoV-2 isolates demonstrated a mix of Alpha (13%), Iota (22%), and B lineages (61%) in Wave 2, predominance of Delta (87.4%) in Wave 3, predominance of Omicron (BA.1) (81%) in Wave 4, and predominance of BA.2 (84%) in Wave 5, with no difference in distribution between adults and pediatrics over time (Figure 5).

**Figure 1.**
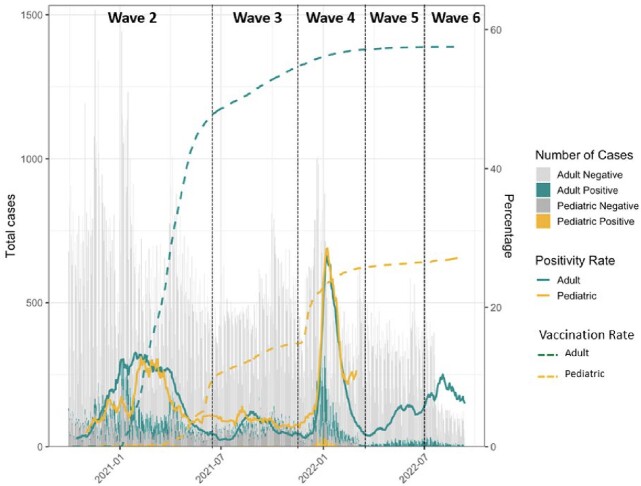
Distribution of SARS-CoV-2 PCR testing, positive tests, positivity rate, and vaccination rate in children and adults.

Number of tested and positive cases presented as daily case counts. Positivity rates presented as 14-day rolling averages. Due to low testing numbers, rolling average of pediatric positivity rate was excluded in Waves 5 and 6. Vaccination rates indicate daily proportion of patients with 2 or more vaccines doses.

**Figure 2.**
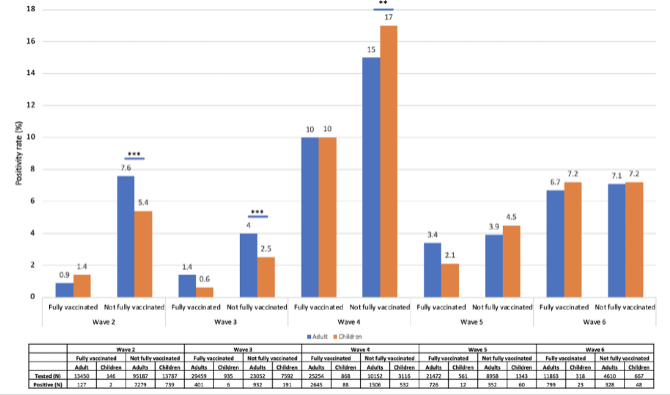
SARS-CoV-2 positivity rates in adults and children by pandemic wave and vaccine status. Positivity rates between fully vaccinated adults and children, and not fully vaccinated adults and children compared within each wave using Pearson’s Chi-squared test. Total number tested and positive for SARS-CoV-2 indicated in table. **p<0.01, ***p<0.001.

**Figure 3.**
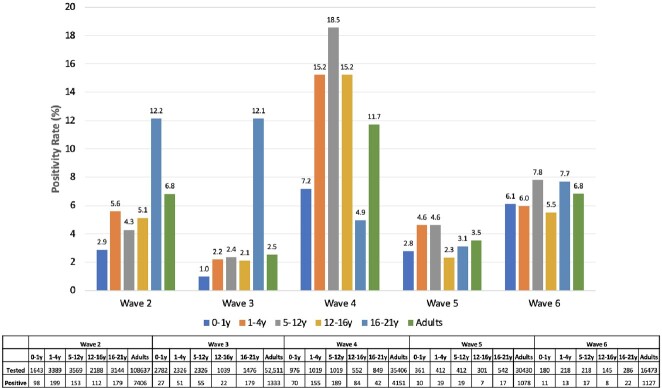
SARS-CoV-2 positivity rates by age group and pandemic wave. Total number tested and positive for SARS-CoV-2 indicated in table.

**Conclusion:**

Despite multiple wave-specific differences, SARS-CoV-2 variant distribution did not differ between adults and children over time. Additional work is indicated to understand if the difference in positivity rates is related to differences in immune response or exposure patterns between children and adults.

**Figure 4.**
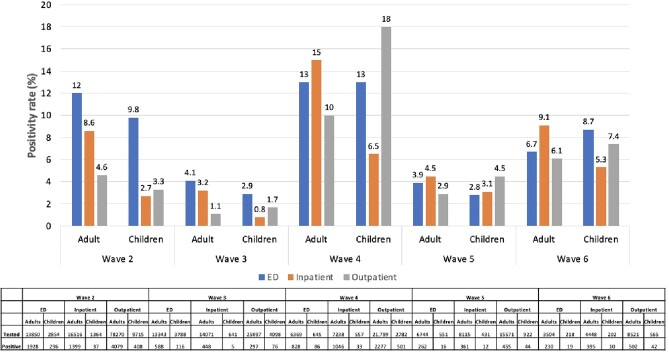
SARS-CoV-2 positivity rates in adults and children by testing site. Total number tested and positive for SARS-CoV-2 indicated in table. ED, emergency department.

**Figure 5.**
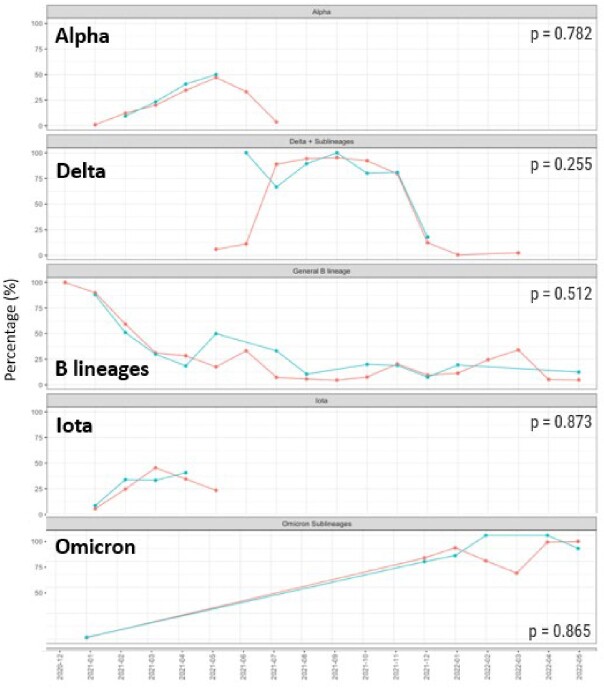
Monthly distribution of SARS-CoV-2 variants in adults and children during Waves 2, 3, 4, and 5. Proportion of each SARS-CoV-2 variant in adults (red line) and children (blue line) over time. Variants indicated by WHO classification and include following Pango lineages: Delta (B.1.617.2, AY), Omicron (BA.1, BA.2), Alpha (B.1.1.7), Iota (B.1.526), B lineages (B.1/B1.1 lineages). The distribution of each variant was compared between adults and children over time using the Kolmogorov-Smirnov test.

**Disclosures:**

**Melissa Cushing, MD**, Cerus Corporation: Advisor/Consultant|Haemonetics: Advisor/Consultant|Octapharma: Advisor/Consultant

